# Response to ‘Reducing plastic in single-use central line insertion packs: a mixed methods observational study’

**DOI:** 10.1177/0310057X261415904

**Published:** 2026-05-26

**Authors:** Ismail K Zazay, James R Burmeister

**Affiliations:** 1 John Sealy School of Medicine, University of Texas Medical Branch, Galveston, USA; 2 Corewell Health William Beaumont University Hospital, Royal Oak, Michigan, USA

We commend Seville et al.^1^ for their mixed-methods study on plastic minimisation within single-use central line insertion packs. The authors clarify clinicians’ ethical distress regarding procedural waste and quantify reductions in attainable carbon emissions and cost through the innovative design of packs. Their results serve to position clinicians as environmental guardians, demonstrating that sustainability can be conceived from the clinical floor and not administrative decree.

While notable, such packs are a temporary fix rather than an enduring one. The greater imperative is to use reusable systems based on sound life cycle analyses (LCAs). McGain et al.^2^ showed that reusable central-line kits emitted more carbon where coal-dominant energy grids prevail. However, Hemberg et al.^3^ later found much lower emissions in renewable-dependent regions. These contradictory findings emphasise that environmental gain is not universal but variable because it depends on local energy profiles, sterilisation efficiency, and material composition.

Substantive challenges to implementation remain. Infection control standards, sterilisation capacity, and procurement systems lag behind the evidence. Davies et al.^4^ observed institutional resistance and perceived safety issues as primary disincentives to reusables despite a growing body of evidence for their feasibility. Lasting and substantive change will therefore need to be inspired by concerted governance that synthesises top-down policy reform with bottom-up clinician initiative.

Seville et al.^1^ provide us with a timely and useful basis. Future research needs to couple environmental and cost data with procurement policy change, standardising LCA reporting within hospital procurement processes so that sustainability is built into healthcare delivery rather than treated as a discrete initiative.
